# Chemical, In Cellulo, and In Silico Characterization of the Aminocholine Analogs of VG

**DOI:** 10.3390/ijms252312656

**Published:** 2024-11-25

**Authors:** Stavroula Kostoudi, Nikolaos Iatridis, Dimitra Hadjipavlou-Litina, Eleni Pontiki, Georgios Pampalakis

**Affiliations:** 1 Laboratory of Pharmaceutical Chemistry, School of Pharmacy, Aristotle University of Thessaloniki, 54124 Thessaloniki, Greece; skostoud@pharm.auth.gr (S.K.); hadjipav@pharm.auth.gr (D.H.-L.); epontiki@pharm.auth.gr (E.P.); 2 Laboratory of Pharmacology, School of Pharmacy, Aristotle University of Thessaloniki, 54124 Thessaloniki, Greece; iatridisn@pharm.auth.gr

**Keywords:** VG, aminocholines, anticholinesterases, SH-SY5Y

## Abstract

V-type nerve agents are exceedingly toxic chemical warfare agents that irreversibly inhibit acetylcholinesterase (AChE), leading to acetylcholine accumulation in synapses and the disruption of neurotransmission. VG or O.O-diethyl S-(diethylamino)ethyl phosphorothiolate was the first compound of this class that was synthesized. The selenocholines (-Se-), cholines (-O-), and methylene-cholines (-CH_2_-) analogs of V-agents have been synthesized and their anti-AChE activities reported. Nevertheless, the aminocholine derivatives have not been pursued. Here, we have designed and synthesized a series of phosphorylated aminocholines analogs of VG that were characterized by NMR spectroscopy (H1, C13, P31, and TOCSY). Their pharmacological properties were analyzed in silico, while their toxicological properties were in vitro investigated using the SH-SY5Y cellular model. Despite the drug likeness of the new compounds, these fail to inhibit AChE in vitro and in cellulo. This may be partially explained by the fact that aminocholine is not a good leaving group compared to thiocholine. Remarkably, one of the compounds (**P4**) was found to even increase the activity of AChE. These compounds may serve as new nerve agent mimics that are safer alternatives for testing countermeasures. Importantly, **P4** may act as a lead compound for developing a new class of alternative nerve agent pretreatments that are safer from pyridostigmine.

## 1. Introduction

**Figure 1:**
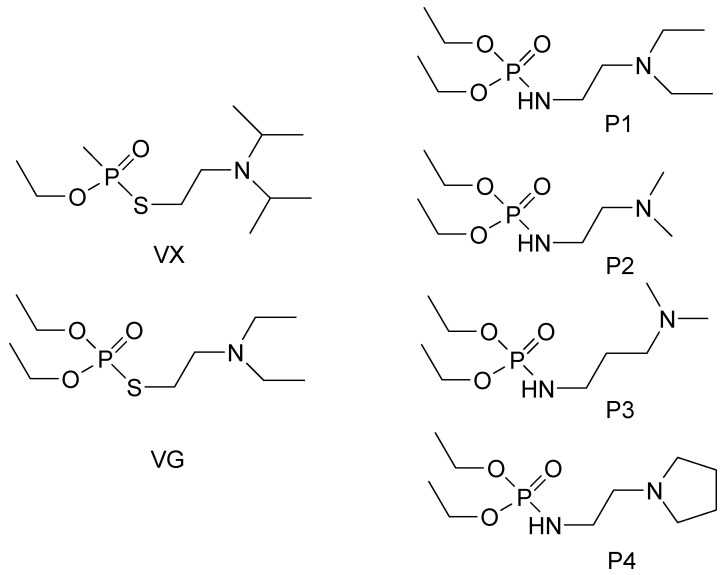
Chemical structures of VX, VG (**left**), the synthesized compounds (**P1**–**P4**) (**right**).

VG [O,O-diethyl S-(diethylamino)ethyl phosphorothiolate], also known as Amiton, is a highly toxic organophosphate nerve agent. More specifically, it is a member of the V-type nerve agents that also includes VX (Figure 1). It was synthesized in 1952 by Ghosh of the Imperial Chemical Industries (ICIs) to be used as an insecticide. Nevertheless, its high toxicity made it too dangerous to be used in agriculture and, thus, it was rapidly withdrawn and prohibited [1]. It is now included in the Schedule 2 chemicals of the Chemical Weapons Convention (CWC). As with nerve agents in general, VG is a potent anticholinesterase agent that rapidly phosphorylates the active site Ser of the acetylcholinesterase (AChE), the enzyme that hydrolyses the neurotransmitter acetylcholine, resulting in irreversible inactivation of the enzyme. The following massive concentration of acetylcholine in neuromuscular junctions causes muscular paralysis, convulsions, and asphyxiation [2].

Understanding the properties of chemical warfare agents as well as their potential derivatives bearing substitutions in certain moieties or atoms is of major importance for assessing potential chemical threats, as these derivatives may exhibit increased toxicity and, at the same time, may not be covered by the CWC. Previously, it was reported that the substitution of the S atom in the molecule of VG with Se yields the selenothiocholine analog of VG that exhibits greater toxicity but displays long-term instability when exposed to air and releases Se [3]. On the other hand, the substitution of S with O renders the phosphor(r/n)ylated choline compounds that are almost non-toxic. Specifically, these compounds exhibit reversible binding to AChE, probably due to the absence of a good leaving group [4]. For example, the O-analog of VX, VO [O-ethyl O-(diisopropylamino)ethyl methylphosphonate], exhibits an LD50 (i.v. mice) of 204,000 μg·kg^−1^ (PUBCHEM: https://pubchem.ncbi.nlm.nih.gov/compound/51437; accessed on 19 July 2024) compared to VX, which shows an LD50 of only 14.5 μg·kg^−1^ [5]. In the same direction, the substitution of the S atom with -CH_2_- in VX results in a compound that exhibits an IC_50_ for AChE 2700 nM compared to 0.3 nM for VX, which is 9000-fold higher [6]. Compounds that exhibit very low toxicity may be used as new lead compounds for the development of new therapeutic agents for glaucoma, Alzheimer’s disease, and myasthenia gravis, or as pretreatments against potential nerve agent exposure. Further, the compounds that better resemble the V-agents may be applied as nerve agent simulants to facilitate the study of agent dissemination, countermeasures, and decontamination as opposed to the “standard” diethyl chlorophosphate that is structurally very different [7,8].

To this end, the compounds derived from the substitution of S with N in the V-type nerve agents have not been produced and investigated for their anticholinesterase action. Thus, in the present study, we have synthesized the aminocholine analogs of VG and examined their ability to inhibit AChE. Interestingly, these compounds failed to inhibit AChE activity in two different assays. On the contrary, the compounds were found to stimulate the activity of AChE.

## 2. Results

### 2.1. Chemistry

The aminocholine derivatives of VG were designed by replacing the -S- atom with the -NH- group. Further, different substituents on the amino group were introduced and, specifically, dimethyl, diethyl, and pyrrolidinyl, in addition to a homocholine analog (Figure 1).

**Scheme 1:**
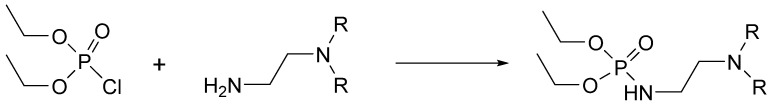
Chemical synthesis of the compounds (**P1**–**P4**).

The compounds were synthesized through a reaction between the diethylchlorophosphate and the corresponding amine, as shown in Scheme 1. The reaction was carried out in the presence of triethylamine and DMF. Attempts to synthesize the compounds in the absence of DMF resulted in substantially lower yields (no more than 7%).

The NMR spectra for the synthesized compounds are as follows.

Diethyl (2-diethylamino)ethyl phosphoramidate (**P1**) (yellow oily liquid, yield: 46%)

^1^H-NMR (CDCl_3_, 500 MHz, ppm): 0.96 (t), 1.28 (dd), 2.47 (m), 2.89 (dq), 3.37 (s, NH) 4.01 (m); ^13^C-NMR (CDCl_3_, 125 MHz, ppm): 11.84, 16.28, 38.74, 46.69, 53.31, 62.15; DEPT-135 (CDCl_3_, 125 MHz, ppm): positive (11.73, 16.22), negative (38.63, 46.58, 53.20, 62.04); ^31^P-NMR (DMSO-d_6_, 202 MHz, ppm): 9.83; TOCSY: 0.94 → 2.45, 1.26 → 4.02, 2.45 → 0.96, 2.46 → 3.37, 2.47 → 2.88, 2.88 → 3.35, 2.89 → 2.47, 3.37 → 2.47, 3.37 → 2.88, 4.02 →1.28.

Diethyl (2-dimethylamino)ethyl phosphoramidate (**P2**) (yellow oily liquid, yield: 33%).

^1^H-NMR (CDCl_3_, 500 MHz, ppm): 1.31 (t), 2.24 (s), 2.41 (t), 2.98 (dq), 3.35 (s, NH), 4.05 (m); ^13^C-NMR (CDCl_3_, 125 MHz, ppm): 16.38, 38.56, 45.11, 59.73, 62.45; DEPT-135 (CDCl_3_, 125 MHz, ppm): positive (16.28, 38.38), negative (44.95, 59.53, 62.59); ^31^P-NMR (DMSO-d_6_, 202 MHz, ppm): 9.31; TOCSY: 1.28 → 4.13, 2.28, 2.42 → 3.01, 3.02 → 2.46, 4.06 → 1.32.

3p. Diethyl (2-dimethylamino)propyl phosphoramidate (**P3**) (yellow oily liquid, yield: 25%).

^1^H-NMR (CDCl_3_, 500 MHz, ppm): 1.32 (t), 1.66 (m), 2.24 (s), 2.40 (t), 2.99 (s), 3.58 (s, NH), 4.04 (m); ^13^C-NMR (CDCl_3_, 125 MHz, ppm): 16.30, 28.51, 40.74, 45.49, 58.17, 62.26; DEPT-135 (CDCl_3_, 126 MHz, ppm): positive (16.22, 45.39), negative (28.30, 40.71, 57.92, 62.11); ^31^P-NMR (DMSO-d_6_, 202 MHz, ppm): 9.00; TOCSY: 1.30 → 4.05, 1.66 → 2.38, 1.66 → 2.99, 2.2, 2.40 → 1.65, 2.40 → 2.98, 2.99 → 1.65, 2.99 → 2.39, 4.06 → 1.31.

Diethyl 2-(pyrrolidine-1-yl)ethyl phosphoramidate (**P4**) (yellow oily liquid, yield: 88%).

^1^H-NMR (CDCl_3_, 500 MHz, ppm): 1.32 (t), 1.78 (m), 2.58 (dd), 3.02 (dq), 3.36 (s, NH), 4.06 (m); ^13^C-NMR (CDCl_3_, 125 MHz, ppm): 16.25, 23.51, 39.74, 53.80, 56.38, 62.24; DEPT-135 (CDCl_3_, 126 MHz, ppm): positive (16.21), negative (23.42, 39.69, 53.76, 56.21, 62.10); ^31^P-NMR (DMSO-d_6_, 202 MHz, ppm): 9.34; TOCSY: 1.30 → 4.05, 1.66 → 2.38, 1.66 → 2.99, 2.2, 2.40 → 1.65, 2.40 → 2.98, 2.99 → 1.65, 2.99 → 2.39, 4.06 → 1.31.

**Figure 2:**
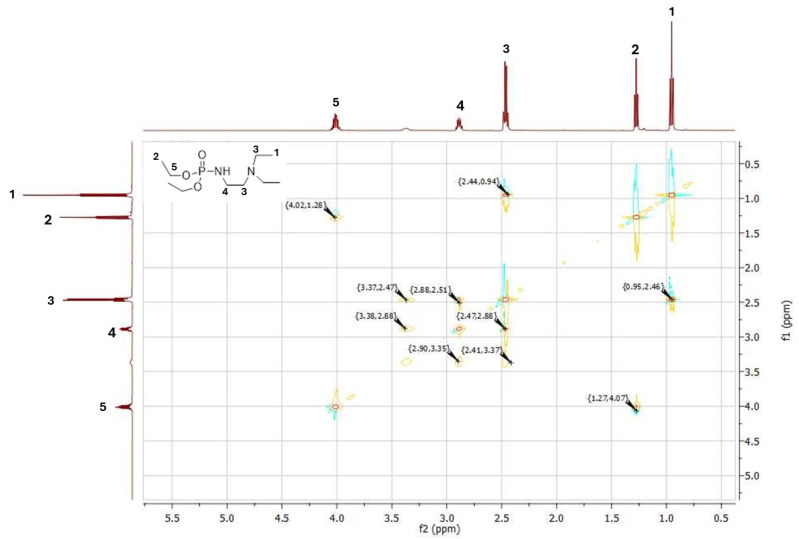
TOCSY-NMR spectrum of compound **P1**.

A representative TOCSY spectrum for the **P1** compound is shown in Figure 2. The spectra for the compounds **P1**–**P4** can be found in the Supplementary Figure.

### 2.2. Druglikeness of the Synthesized Compounds

The drugability of the derivatives was carried out in silico as described in the Section 4. The data depicted in Table 1 indicate that all compounds fulfill the Lipinski rule of five (MW ≤ 500, logP_o/w_ ≤ 5, H-acceptors ≤ 10, H-donors ≤ 5), the Ghose rule [160 ≤ MW ≤ 480, −0.4 ≤ logP_o/w_ ≤ 5.6, 40 ≤ MR ≤ 130, 20 ≤ number of atoms ≤ 70], the Veber filter [number of rotatable bonds ≤ 10, topological polar surface area (TPSA) ≤ 140 Å^2^], the Egan rule (logP_o/w_ ≤ 5.88, TPSA ≤ 131.6 Å^2^) [9], and the Muegge filter (200 ≤ MW ≤ 600, −2 ≤ logP_o/w_ ≤ 5, TPSA ≤ 150 Å, number of rings ≤ 7, number of carbon atoms > 4, number of heteroatoms > 1, number of rotatable bonds ≤ 15, H-acceptors ≤ 10, H-donors ≤ 5) [10] for pharmacologically active molecules. Further, the compounds are predicted to penetrate both the gastrointestinal tract and the blood–brain barrier (BBB). Also, the compounds showed a sp^3^ fraction (related to solubility) of 1, which is ≥0.42, which is considered suitable for pharmacologically active compounds. Moreover, they display an unbound fraction (Fu) ≥ 87, indicating that these compounds are expected to remain free in plasma; therefore, they can diffuse more efficiently. **P2** and **P3** compounds are predicted to exhibit excellent gastrointestinal permeability, which is an important feature of orally administered drugs. Finally, all compounds display optimal volume distribution (VD) for pharmacologically active molecules.

### 2.3. Molecular Docking

AChE obtained from *Εlectrophorus electricus* (eeAChE) was selected for the biological assay, as it is a widely used enzyme in assays encompassing the testing of AChE inhibitors [12,13]. eeAChE shares structural homology with human AChE (hAChE) and from other organisms [14]. The UniProt web database [15] was used to retrieve the complete protein sequence for eeAChE as a FASTA sequence format. Running an alignment of the FASTA sequence format through UniProt with the Clustal Omega program of the eeAChE shows 60.4% identity, 74.2% positives, 633 query length, and 614 match length with AChE from *Homo sapiens*.

Homology modeling was performed to generate the 3D structure of eeAChE. Molecular docking was performed to all the novel derivatives to the four optimized complexes derived from the alignment of the I-TASSER-generated 3D model of AChE with experimental 3D protein structures of human origin from the Protein Data Bank (PDB) (4BDT.A, 4EY5.A, 4EY6.A, and 4EY7.A) and subsequent minimization.

**Figure 3:**
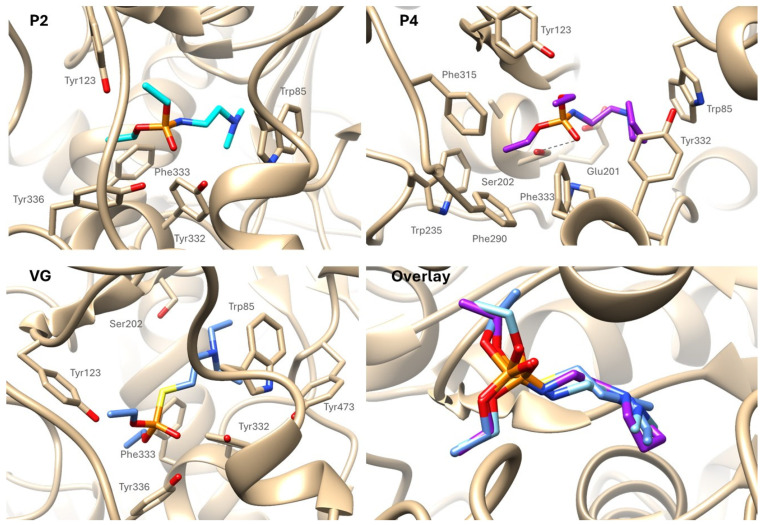
Best docking pose of compounds **P2** (cyan), **P4** (purple), and VG (light blue) with the 3D model of eeAChE generated based on the 4EY6 human structure deposited in the PDB. The one hydrogen bond is illustrated with dashed grey lines. The heteroatoms are colored differently: nitrogen (blue), oxygen (red), sulfur (yellow) and phosphorus (orange).

Compounds **P2** and **P4** presenting the best docking scores seem to bind in the same manner as the reference compound VG. Compound **P2** develops hydrophobic interactions with Tyr123, Tyr332, Phe333, and Tyr336, and π-cation interactions with Trp85, extending into the anionic site, the acyl binding site, and the peripheral anionic site. **P4** develops hydrophobic interactions with Trp85, Tyr123, Trp235, Phe290, Phe333, Tyr332, and Phe333; Hydrogen bonds with Ser202, π-cation interacts with Trp85, and there is a salt bridge with Glu201 for the catalytic, anionic, acyl binding, and peripheral anionic site. VG being used as a reference compound interacts with the enzyme hydrophobically with Trp85, Tyr123, Tyr332, Phe333, Tyr336, and Tyr473 and through π-cation interactions with Trp85 through the anionic, acyl binding, and peripheral anionic site (Figure 3).

### 2.4. Inhibition of AChE Activity

**Figure 4:**
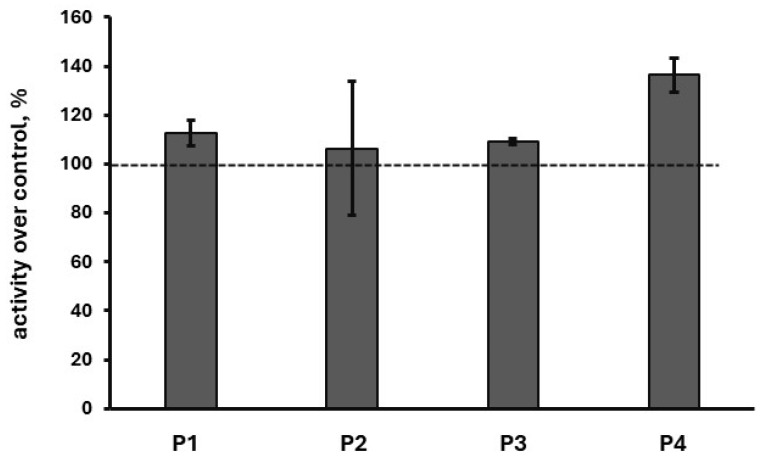
Activity of eeAChE in the presence of compounds **P1**–**P4**. None of the compounds inhibited the activity of eeAChE. On the contrary, stimulation of eeAChE activity was observed mainly for compounds **P1** and **P4**. All compounds were tested at 100 μM.

The compounds were incubated with *Electrophorus electricus* AChE (eeAChE). eeAChE has been widely used in experiments to determine the inhibitory capacity of organophosphates or test reactivation mechanisms and drugs [12,13]. Although the docking suggested that compounds **P2** and **P4** bind in the same manner as VG to the active site, all compounds failed to inhibit the activity of eeAChE (Figure 4). This may be related to the fact that after VG enters the enzyme’s active site, it readily phosphorylates the active site Ser and renders the enzyme inactive, whilst the synthesized compounds contain the bad leaving group aminocholine that does not permit phosphorylation and permanent inactivation of AChE. Notably, an increase in eeAChE activity was observed in the presence of the compounds. This action is likely linked to an allosteric effect.

### 2.5. In Cellulo Assessment of the Compounds in SH-SY5Y Cells

Variations between human AChE and eeAChE were recently highlighted and pointed to the necessity to study the inhibition of AChE in a humanized in vitro assay [12]. As a cellular model, we used the neuroblastoma SH-SY5Y cells that can be induced to differentiate into neuron-like cells with retinoic acid. Further, the same system allows the simultaneous assessment of BChE inhibition.

**Figure 5:**
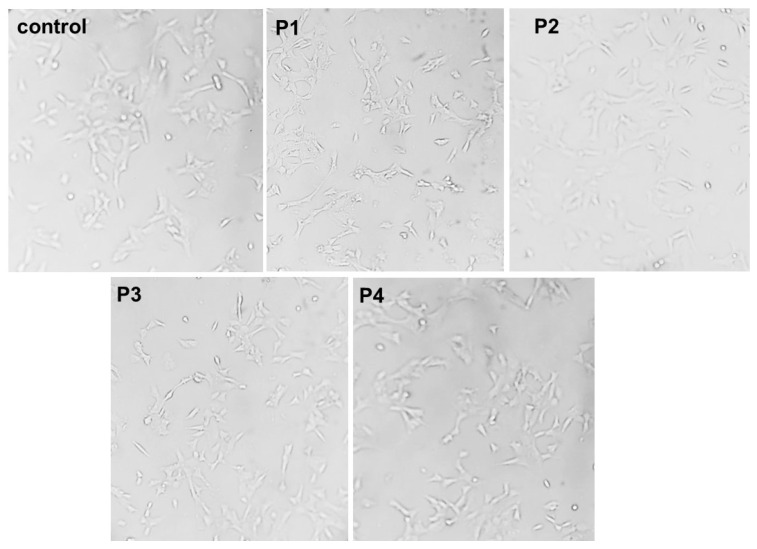
SHSY-5Y after 24 h incubation with inhibitors **P1**–**P4** at 50 μM (40× magnification). No alterations in the morphology of SHSY-5Y cells were observed.

**Figure 6:**
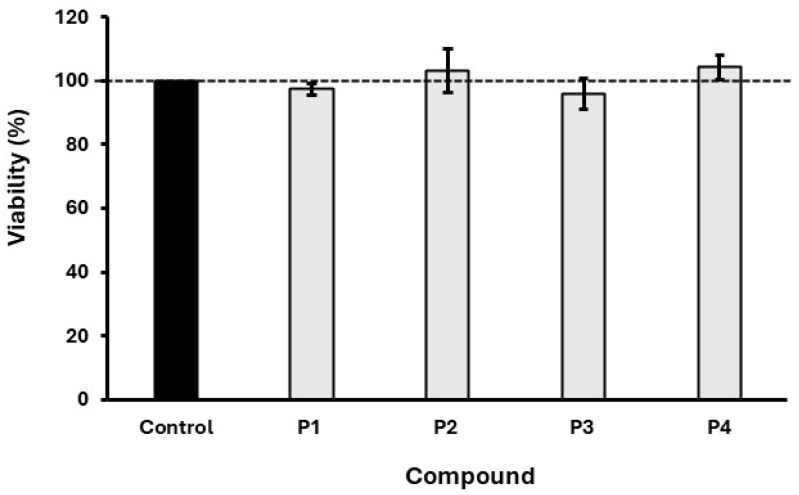
The **P1**–**P4** compounds did not exhibit cytotoxicity in SH-SY5Y cells. The results shown are from an MTT assay carried out 24 h after the incubation of cells with the **P1**–**P4** compounds at a concentration of 50 μM. The results are shown ± standard deviation.

Initially, 50 μM of **P1**–**P4** compounds were incubated with the cells to verify that these compounds do not exhibit any cytotoxicity. Indeed, as shown in Figure 5 and Figure 6, no signs of cytotoxicity were observed either morphologically under the microscopy or with the MTT assay, respectively. Once it was proven that these compounds do not show any cytotoxicity, we proceeded to analyze their effect on AChE activity. To this end, it should be noted that even VX does not show cytotoxic action in SH-SY5Y cells (IC_50_ 1100 μM) [16]. This is important since the potential cytotoxic effects of **P1**–**P4** to SH-SY5Y cells could lead to decreased cell numbers, which, in turn, will result in artificially decreased activities of AChE.

**Figure 7:**
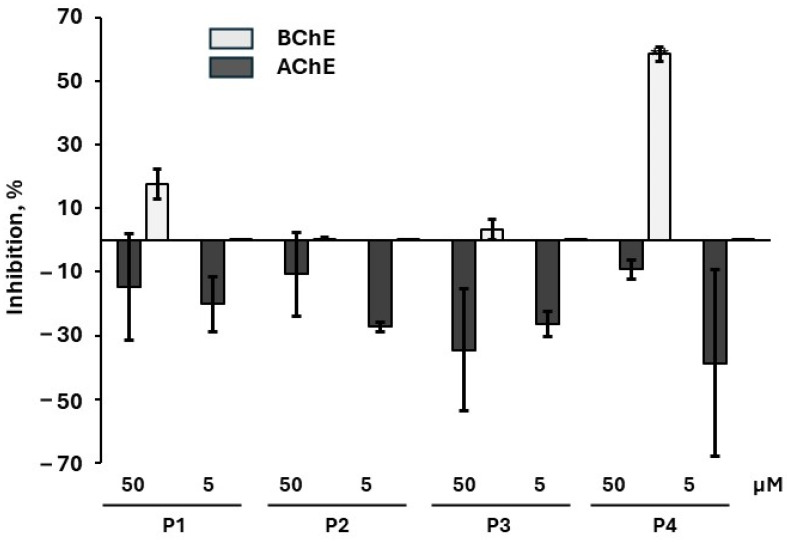
AchE (dark bars) and BChE (light gray bars) inhibition by compounds **P1**–**P4**. The compounds fail to inhibit AChE but, interestingly, compounds **P1** and **P4** inhibit BChE at 50 μM. Especially, compound **P4** shows approximately 60% inhibition. Furthermore, all compounds appear to stimulate the activity of AChE (negative values of inhibition). The results are shown ± standard deviation.

For the enzymatic assay, the compounds were incubated with the SH-SY5Y cells at concentrations of 50 and 5 μM. As shown in Figure 7, compounds **P1**–**P4** fail to inhibit AChE in the cellular assay consistently, with the experiments carried out with purified protein. On the contrary, compound **P4** showed a concentration-dependent activation of AChE. The other compounds, **P1**–**P3**, also acted as stimulators of AChE activity without, however, exhibiting a concentration-dependent effect. In addition, we have studied the inhibition of BChE by compounds **P1**–**P4** in SH-S5Y5Y cells. Remarkably, **P4** was found to inhibit BChE when applied at 50 μM.

**Figure 8:**
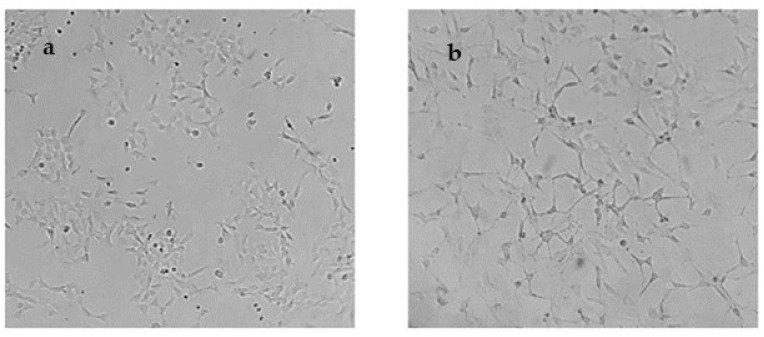
Differentiation of SH-SY5Y cells. The SH-SY5Y cells are shown on day 1 (**a**) and day 7 (**b**) of differentiation with 10 μM retinoic acid. The development of neurites is easily detectable.

Then, the cells were differentiated and exposed to **P1**–**P4** compounds as performed previously. Initially, the differentiation of SH-SY5Y cells by retinoic acid was morphologically observed with microscopy, as shown in Figure 8. The differentiated SH-SY5Y cells show an extensive neurite outgrowth and network as well as pyramidal bodies as expected [17].

**Figure 9:**
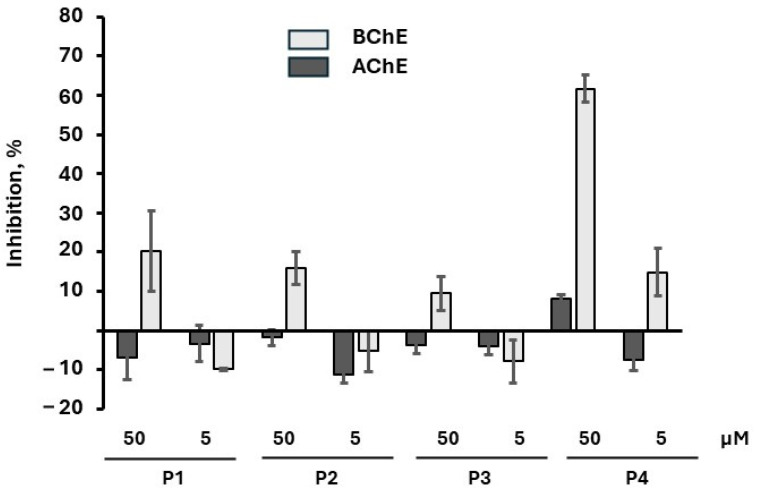
Inhibition of AChE and BChE by **P1**–**P4** in differentiated SH-SY5Y cells. The results are shown ± standard deviation.

Then, compounds **P1**–**P4** were applied to the differentiated SH-SY5Y cells (Figure 9). It was demonstrated that the activity of AChE was not affected, while the activity of BChE was inhibited by the compounds **P1** and **P4** as with undifferentiated cells. This effect was concentration dependent, with 50 μM exhibiting the highest inhibitory action. Further, regarding the stimulation of AChE activity, only **P2** exhibited a dose-dependent response. This indicates that there is no difference in the response to the synthesized compounds between undifferentiated and differentiated cells. Thus, undifferentiated SH-SY5Y can be used for the assessment of AChE activity as well as differentiated.

## 3. Discussion

The investigation of nerve agent analogs is important for the potential identification of new simulants and surrogates. In this direction, the identification of V-agent mimics is very important. Further, the design of V-agent analogs could assist in the elucidation of their mechanism of toxicity. In this direction, the replacement of the -S- atom by selenium was found to increase their toxicity [3], while replacement with O was found to reduce toxicity. Therefore, within the same row in the periodic table, going downwards, an increase in the potency of the agent is observed. On the other hand, the replacement of -S- with methylene [6] significantly reduces the anti-AChE action. Here, we investigated the effect of -S- replacement with -NH-. To our knowledge, only one study has synthesized phenylphosphonate aminocholines derivatives, but there was no examination of their potential anti-AChE action [18].

VG was selected as the parent compound for the design of new agents since (a) it lacks a stereogenic center, thus omitting stereospecific interactions and potentially making the interpretation of data easier and (b) the parent compound is the least toxic agent from the V-series, and although replacement with -NH- is considered to reduce toxicity, it was not clear whether the new compounds would be harmless.

The compounds were synthesized and tested against purified AChE and in the SH-SY5Y cellular system that has been proposed as a model to study potential neurotoxic agents, including nerve agents [16,19,20].

The compounds did not show any signs of cytotoxicity. This is not surprising since even VX or A-234 do not show cytotoxic action in SH-SY5Y cells (IC50 1100 μM and 12,000 μM, respectively) [16]. To this end, it should be noted that in animals, the toxicity of VX is due to AChE inhibition, causing a massive accumulation of acetylcholine that results in muscular paralysis, including paralysis of respiratory muscles, and, eventually, respiratory failure. On the other hand, no such mechanism is present at the cellular level, and, therefore, the inhibition of AChE in SH-SY5Y by VX is expected to be innocuous. Further, the absence of cytotoxic action by VX indicates that VX is not a cytotoxic agent.

All compounds were tested both in differentiated and undifferentiated SH-SY5Y cells. There were no significant changes between the results obtained between differentiated and undifferentiated cells. No inhibition of AChE was detected while stimulation of its activity was observed. It is unlikely that the stimulation of AChE activity occurs through **P1**–**P4** binding to the active site since this binding is expected to prevent the substrate from approaching the active site. Thus, it is more likely that another allosteric site is affected, which, in turn, stimulates the activity of the enzyme.

The thiono-analogs of **P1**, **P3**, and **P4**, namely, the compounds bearing -O- instead of -NH- and P=S instead of P=O, were synthesized previously and found to inhibit AChE, albeit less effectively than VG [21]. None of the thiono-analogs was shown to stimulate the activity of AChE. Also, none of the thiono-analogs were tested against BChE to determine whether they could inhibit BChE.

Interestingly, compound P4 was found to inhibit BChE. The basis for this inhibition is currently unknown but merits future investigation. Although we did not measure the LD_50_ of the **P1**–**P4** compounds, the absence of anti-AChE action, even at 100 μM, likely suggests that these compounds are not toxic. This resembles the O-analog of VX, VO that has a reported LD_50_ iv in mice of 204,000 μg∙kg^−1^ relative to 14.5 for VX, which is 14,000 times higher.

In addition, our study further highlights the suitability of the SH-SY5Y cellular assay for testing neurotoxicants. In this direction, the simultaneous occurrence of BChE allowed us to test the inhibition of both AChE and BChE in a single assay. The small variations in the stimulating or inhibitory action of **P1**–**P4** against AChE and BChE in differentiated and undifferentiated cells may be related to the different levels of AChE/BChE expression and activity between these cellular populations. The different expression levels will result in differences in signal acquisition that, in turn, could account for the observed small variations.

Our study set the basis of the development of selective BChE inhibitors using, e.g., compound **P4** as a lead compound. The interest in developing BChE selective inhibitors is based on the fact that BChE inhibitors are suggested as a new approach for the treatment of Alzheimer’s disease [22]. Also, the ability of these compounds to act as AChE stimulators indicates that these compounds could be used as leads for the development of AChE stimulators for effective countermeasures against nerve agent poisoning. Finally, the in silico predicted druglikeness further corroborates their application as lead compounds for pharmacological exploitation towards the above-mentioned directions.

## 4. Materials and Methods

### 4.1. Materials

All chemicals were obtained from Sigma-Aldrich (St. Louis, MO, USA) and were used without further purification.

### 4.2. General Synthesis

The desired organophosphate compounds were obtained through the reaction of diethyl chlorophosphate with the corresponding substituted aminocholine using a modified synthesis procedure [23]. Specifically, in a three-necked round bottom flask under an Ar atmosphere, anhydrous DCM (approximately 4 mL) and a catalytic amount of DMF (1 to 2 mL) were added. Then, Et_3_N (1 eq), along with the corresponding amine (1 eq), were added. The mixture was cooled just below 0 °C, and diethyl chlorophosphate (1 eq) was added dropwise and carefully. The above mixture reaction after incubation for 48 h at room temperature was extracted with brine to obtain a yellow oil that was subjected to detailed NMR spectroscopic analysis. No further purification was needed.

### 4.3. NMR Spectroscopy

A 500 MHz NMR spectrometer (Agilent, Santa Clara, CA, USA) was employed for ^1^H, ^13^C, and ^31^P NMR experiments. Additionally, 2D (TOCSY-NMR) and DEPT-135 spectra were obtained. All the spectra were recorded at ambient temperature using CDCl_3_ as solvent, except for ^31^P that was obtained in DMSO-d6.

### 4.4. Purified AChE Activity

The ability of the compounds to inhibit AChE was determined in vitro with Ellman’s assay [24,25,26]. For AChE, the *Electrophorus electricus* AChE (eeAChE) was used (Sigma-Aldrich). Initially, stock solutions of the organophosphate compounds were prepared in DMSO (final concentration of 10 mM). The assay was performed in PBS pH 8 containing 3.5 U/mL AChE. The reaction mixture contained the compounds at the final concentration of 0.1 mM, the substrate acetylthiocholine iodide at 0.16 mM, and 0.2 mM yellow dye 5,5′-dithiobis (2-nitrobenzoic acid) (DNTB). AChE hydrolyzes acetylthiocholine to thiocholine, which, in turn, reacts with DNTB to yield a product that absorbs at 412 nm. The absorbance was measured on the Thermo electron corporation Heλιος γ device. In the enzymatic reactions, the final concentration of DMS did not exceed 1%.

### 4.5. Cell Line and Differentiation

The potential bioactivity of the synthesized compounds was also tested with the neuroblastoma SH-SY5Y cells. These cells were previously used in neurotoxicological studies involving nerve agents [16] and anticholinesterase pesticides [19]. The SH-SY5Y (ATCC) were cultivated in Dulbecco’s Modified Eagle’s medium, high glucose, with Sodium Pyruvate (DMEM; Biosera, Cholet, France) supplemented with 10% fetal bovine serum (FBS; PAN Biotech, Aidenbach, Germany) and a 1% Penicillin–Streptomycin antibiotic mixture (P/S; Biosera). Cells were cultivated at 37 °C and 5% CO_2_ in a humidified incubator. Cells were regularly split at 80–90% confluence. The assay was conducted in both undifferentiated and differentiated SH-SY5Y cells.

For experiments in non-differentiated SHSY-5Y cells in 96-well plates, 30 × 10^3^ cells were seeded per well. The cells were cultured for one day and then exposed to the compounds for 24 h.

The differentiation of SH-SY5Y cells was induced with retinoic acid. Initially, non-differentiated SHSY-5Y cells were seeded to a 96-well plate (4 × 10^3^ cells per well). The following day, the culture medium was aspirated, and 100 μL of the differentiation medium containing DMEM, 10% FBS, 1% P/S, and 10 μM retinoic acid was added. The differentiation medium was changed every 48 h for a total of seven days. On differentiation day seven, the cells were exposed to the compounds for 24 h.

### 4.6. Determination of AChE and Butyrylcholinesterase Activity in SH-SY5Y Cells

The inhibitory effects of the synthesized compounds were tested on non-differentiated and differentiated SHSY-5Y cells following a modified Ellman’s method as described [16]. Briefly, during the first day of the experiment, the medium was aspirated from each well, and the medium containing each inhibitor was added (50 μM and 5 μM). The cells were incubated with synthetic inhibitors for 24 h. Then, the cells were lysed with a Phosphate Buffer Saline (PBS) solution containing 0.1M DTNB, 2.23 mM acetylthiocholine iodide (AChE substrate) or butyrylthiocholine iodide (BChE substrate), and 1% Triton X-100. A total volume of 90 μL was added to each well and was pipetted gently up and down to lyse the cells. The plate was then covered with foil and incubated at room temperature for 24 h. The following day, the absorbance of each well was measured at 405 nm using the Perkin Elmer 2030 Spectrophotometer to determine enzymatic activity. Enzyme inhibition was calculated compared to controls (cells incubated with DMSO alone).

### 4.7. In Vitro Cytotoxicity Assay

SHSY5Y cells were seeded at a density of 10,000 cells per well on 96-well plates. The following day, the media was aspirated, and treatment was performed with each inhibitor at a final concentration of 50 μM in each well in duplicates. An untreated condition was used as viability control, where the inhibitors were absent, and an equal amount of inhibitor diluent (DMSO) was present. Each experiment was performed three times. MTT was added to each well at a final concentration of 0.5 mg/mL, and the microplate was incubated at 37 °C, 5% CO_2_ for 4 h. The formation of formazan crystals was confirmed by observing the cells on an inverted microscope. The medium was aspirated, and 100 μL of DMSO was pipetted up and down in each well to dissolve the formazan crystals. The plate was then foil covered and incubated at 37 °C, 5% CO_2_ for 30 min. Absorbance was measured at 600 nm using the EL10B Biobase Elisa Microplate Reader. The cell viability under each condition was normalized to the cell viability of the untreated control condition as a percentage value for every experiment.

### 4.8. In Silico Druglikeness Prediction

Three different software online platforms were used, ADMETlab2 (http://admetmesh.scbdd.com, accessed on 9 July 2024) [11], SWISSAdme (http://www.swissadme.ch, accessed on 9 July 2024), and pkCSM (http://biosig.lab.uq.edu.au/pkcsm, accessed on 29 October 2023) [27]. The structure of the compound is entered in a SMILES format or with the appropriate draw tool.

### 4.9. Experimental Protocol for Molecular Docking

As mentioned above, there is no experimental 3D structure of eeAChE in the PDB. The complete protein sequence is obtained from UniProt [15] in FASTA sequence format with accession code O42275. It is a protein with 633 amino acids and a molecular weight of 71,815 Da. As previously reported, the signal peptide responsible for protein translocation comprising 23 amino acid residues is cleaved [14]. Using the amino acids sequence annotated in UniProt, a 3D model was generated with I-TASSER [28,29,30]. The UCSF Chimera MatchMaker procedure was used for aligning the generated 3D model of AChE, with the following 3D protein structures of human origin from the PDB [4BDT.A [31], 4EY5.A, [32] 4EY6.A [32], and 4EY7.A [32]] containing active ligands used as drugs and minimized, taking into account protein flexibility [33]. The aligned PDB structures were removed from the protein part and the ligands were maintained. Therefore, four new complexes were obtained: the 3D-AChE *Electrophorus electricus* with X-ray co-crystallized ligands (4BDT:HUW, 4EY5:HUP, 4EY6:GNT, and 4EY7:E20). Finally, these four new complexes were geometrically optimized by minimization using UCSF-Chimera with an AMBER14SB force field [34,35]. TIP3P was used for the explicit water model and total system charge neutralization with Na^+^Cl^−^ ions. Ligand topologies and parameters were generated with Antechamber [36]. Please add this sentence. The minimization procedure consisted of 100 steepest descent steps with 0.02 Å step size followed by 10 conjugate gradient steps with 0.02 Å step size. The four optimized complexes were used for the cross-docking simulations. Docking simulations were run with the vinardo scoring function [37], as implemented in SMINA [38]. Docking was carried out using a grid box of size 25 Å in the X, Y, and Z dimensions with an exhaustiveness value of 64 and a maximum output of 20 docking modes [39,40,41]. These dimensions of the grid box were selected according to the used crystal structures co-crystallized with known AChE inhibitors because it was necessary to cover not only the catalytic site but also the anionic site, the oxyanion hole, the acyl binding pocket, and the peripheral anionic site of AChE and nearby residues. The docking result’s analysis and visual inspection were carried out using UCFS Chimera [33]. All ligands were docked to the four optimized complexes. The best docked pose was selected considering the docking score since it helps to select the best poses and rank the ligands through a mathematical approximation [42].

## Figures and Tables

**Table 1:** In silico predicted druglikeness for compounds **P1**–**P4**.

	Compound P1			Compound P2			Compound P3			Compound P4		
	ADMET lab2	Swiss ADME	pkCSM	ADMET lab2	Swiss ADME	pkCSM	ADMET lab2	Swiss ADME	pkCSM	ADMET lab2	Swiss ADME	pkCSM
H-acceptor	5	5	4	5	5	4	5	5	4	5	5	4
H-donors	1	1	1	1	1	1	1	1	1	1	1	1
Rotatable bonds	10	10	10	8	8	8	9	9	9	8	8	8
Fraction sp^3^ (sp^3^ hybridized C/total C)	1.0	1.0		1.0	1.0		1.0			1.0	1.0	
Van der Waals volume	249.708			215.116			232.412			241.151		
TPSA	50.8	60.61	99.080	50.8	60.61	86.350	50.8	60.61	92.715	50.8	60.61	98.074
Molecular refractivity (MR)		66.72			57.10			61.91			68.52	
LogP_o/w_	0.51	1.35	2.0989	−0.318	0.71	1.3187	−0.089	1.05	1.7088	0.137	1.10	1.8529
LogD (LogP_o/w_ at pH 7.4)	0.052			−0.619			−0.782			0.188		
LogS (mol·L^−1^) (solubility)	0.42	−1.21 −1.59 −2.75	−1.903	0.856	−0.71 −0.83 −1.94	−1.372	0.841	−0.96 −1.21 −2.35	−1.22	0.429	−1.18 −1.34 −2.27	−2.062
Blood–brain barrier penetration		Yes			Yes			Yes			Yes	
Gastrointestinal penetration		High			High			High			High	
Caco-2 permeability (cm∙s^−1^) *	−4.646			−5.273			−5.331			−4.935		
Skin penetration (cm∙s^−1^)		7.31	6.79 **		−7.66	6.89		−7.49	6.82		−7.47	6.88
Fu (%)	87.794			90.969			91.329			88.762		
VD (L kg^−1^)	0.813			1.151			1.268			0.960		

* Relates to gastrointestinal penetration [excellent > −5.15 [11]], ** the values of pkCSM are given in cm∙h^−1^ and converted to cm∙s^−1^.

## Data Availability

Data are contained within the article. This article is a revised and expanded version of a poster entitled “Synthesis and biological evaluation of V-type nerve agent mimics”, which was presented at the 23rd Panhellenic Chemistry Conference, 25–28 September 2024, National and Kapodistrian University of Athens, Central Building, Greece [43], and as a poster entitled “Design, synthesis of a new class of organophosphate acetylcholinesterase inhibitors for pharmacological applications”, which was presented at the 19th Hellenic Symposium on Medicinal Chemistry (HSMC-19), 9–11 March 2023, Patras, Greece [44].

## Supplementary Materials

The following supporting information can be downloaded at https://www.mdpi.com/article/10.3390/ijms252312656/s1.
