# Supplementary material for: Chemical, In Cellulo, and In Silico Characterization of the Aminocholine Analogs of VG
Source: Int J Mol Sci. 2024 Nov 25;25(23):12656. doi: 10.3390/ijms252312656 (PMC11641387; doi:10.3390/ijms252312656)
Supplement: Supplementary file 1 [file ijms-25-12656-s001.zip › ijms-3314644-supplementary.pdf]

P1

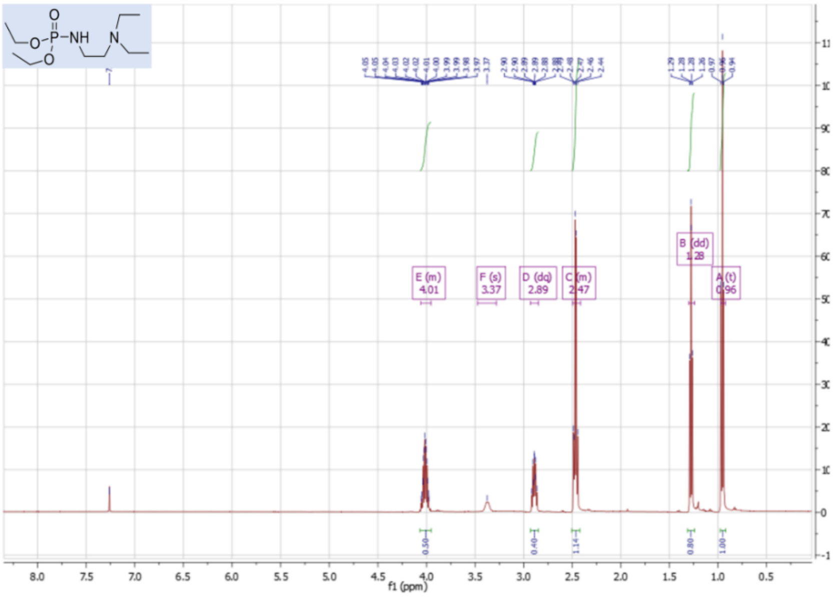

^{1}HNMR spectrum of **P1**

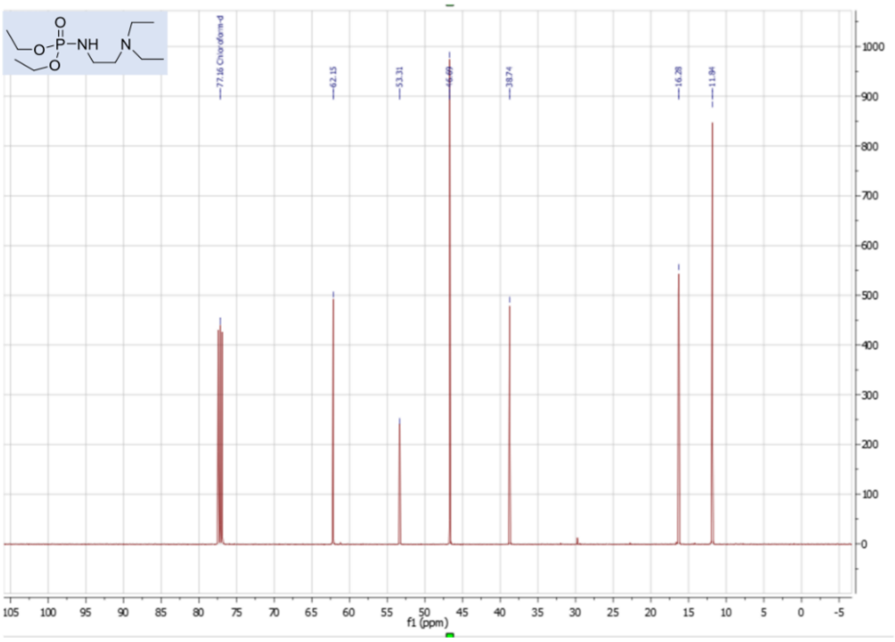

^{13}CNMR spectrum of **P1**

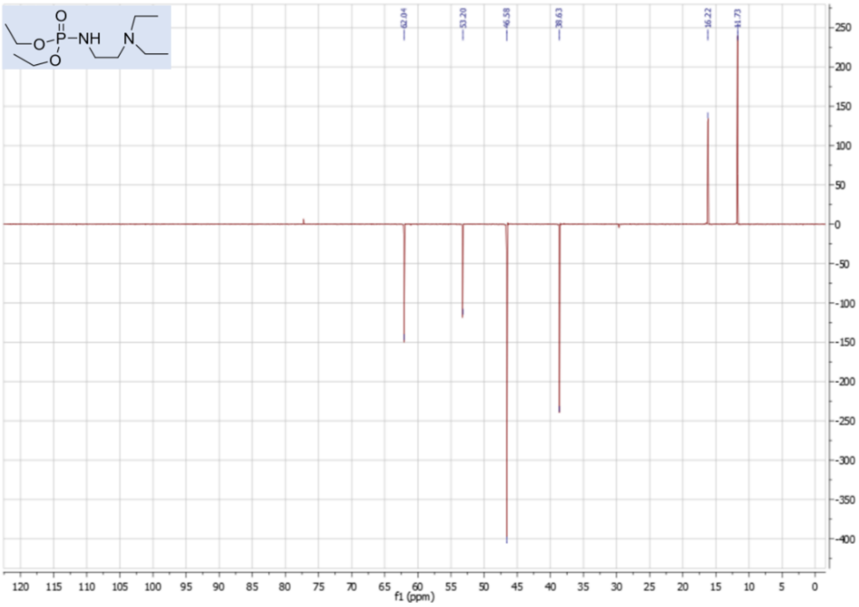

DEPT-135 spectrum of **P1**

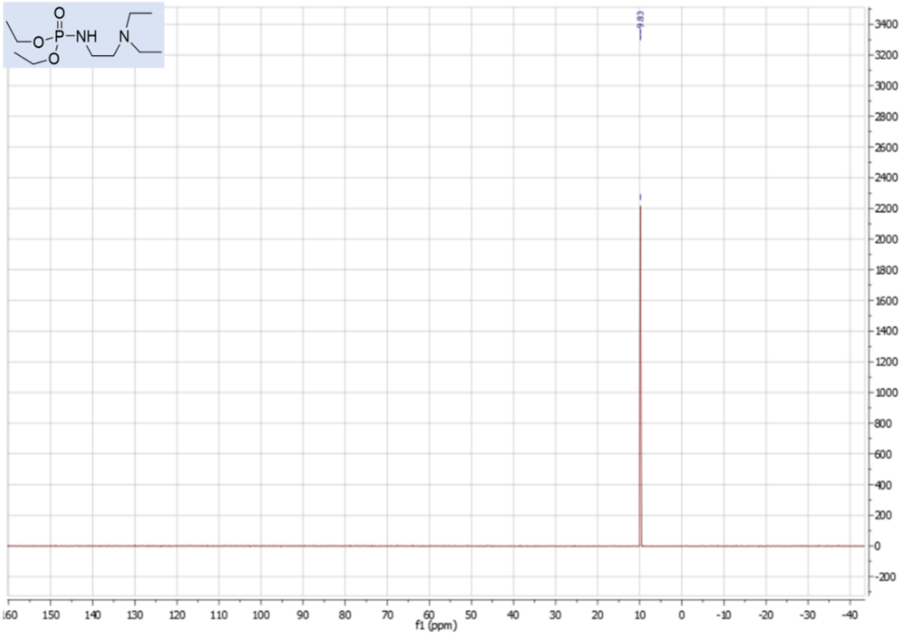

^{31}PNMR spectrum of **P1**

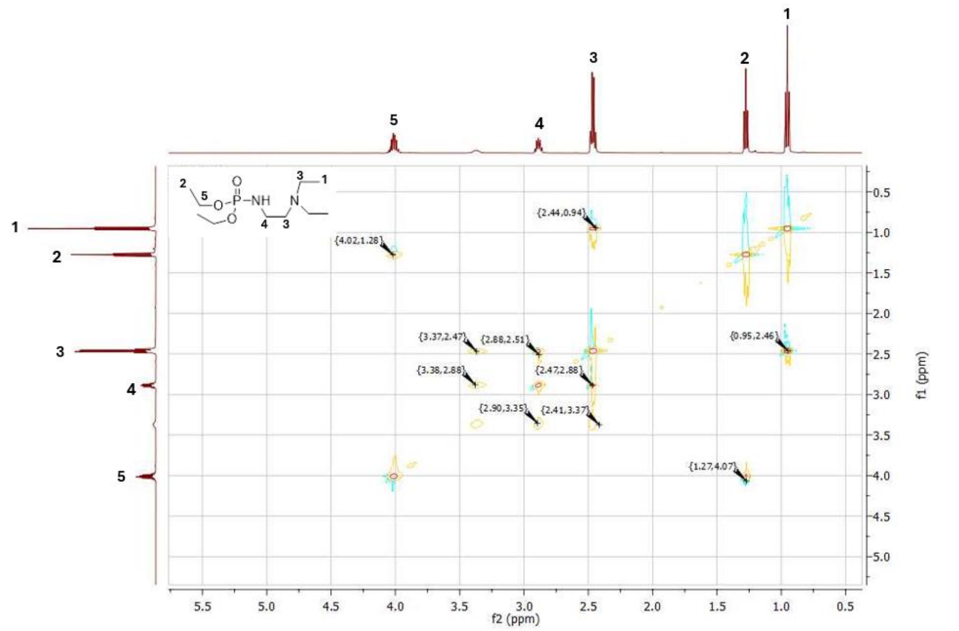

TOCSY spectrum of **P1**

P2

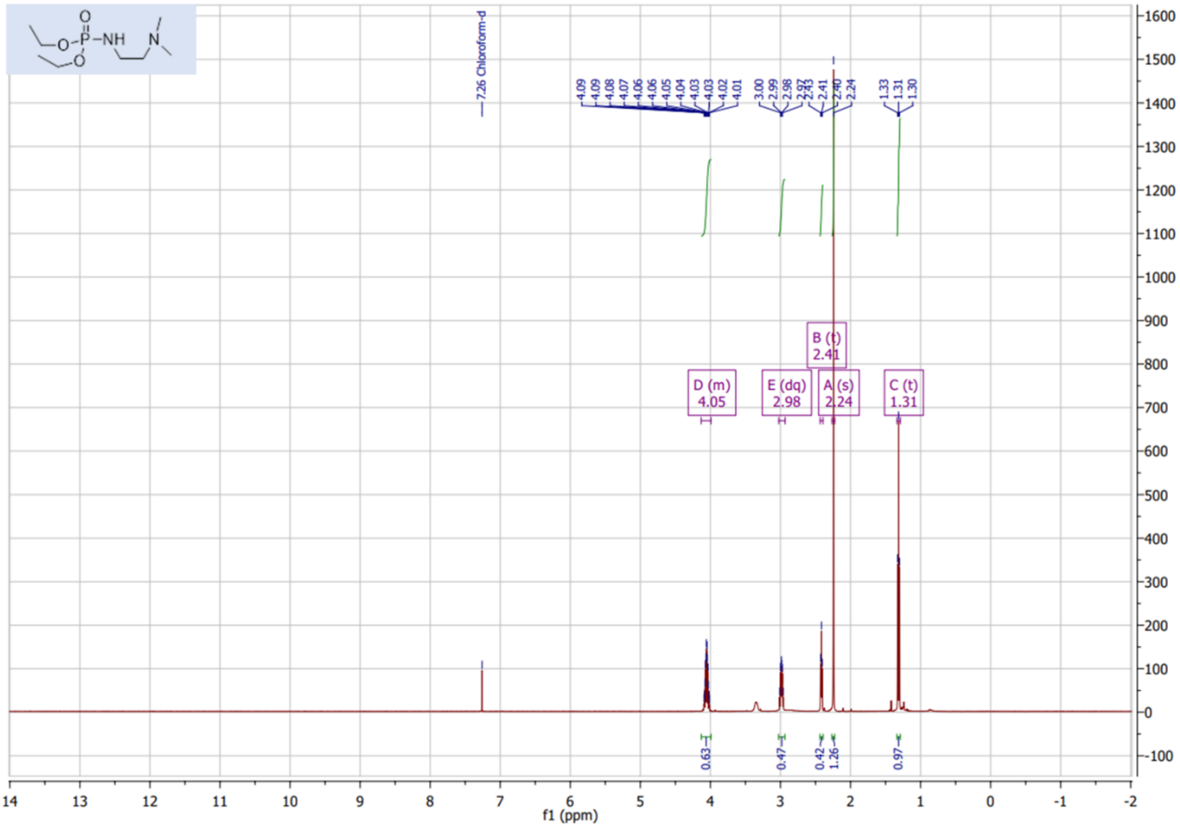

^{1}HNMR spectrum of **P2**

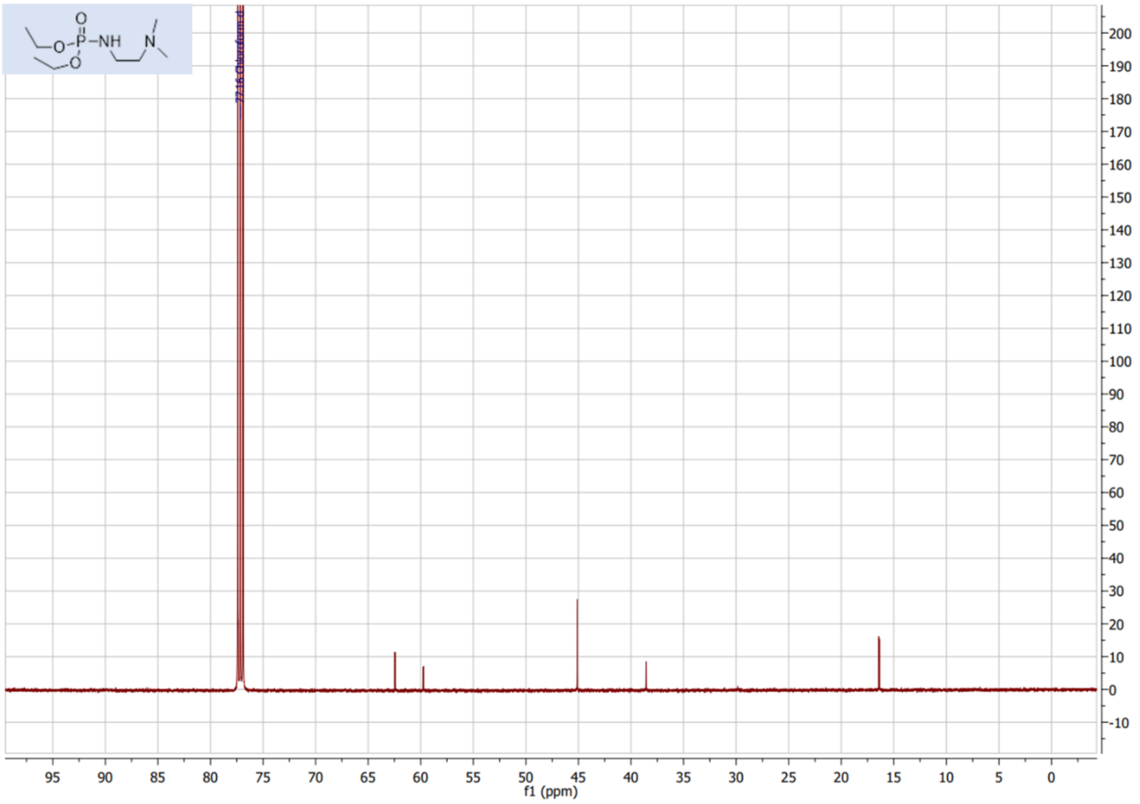

^{13}CNMR spectrum of **P2**

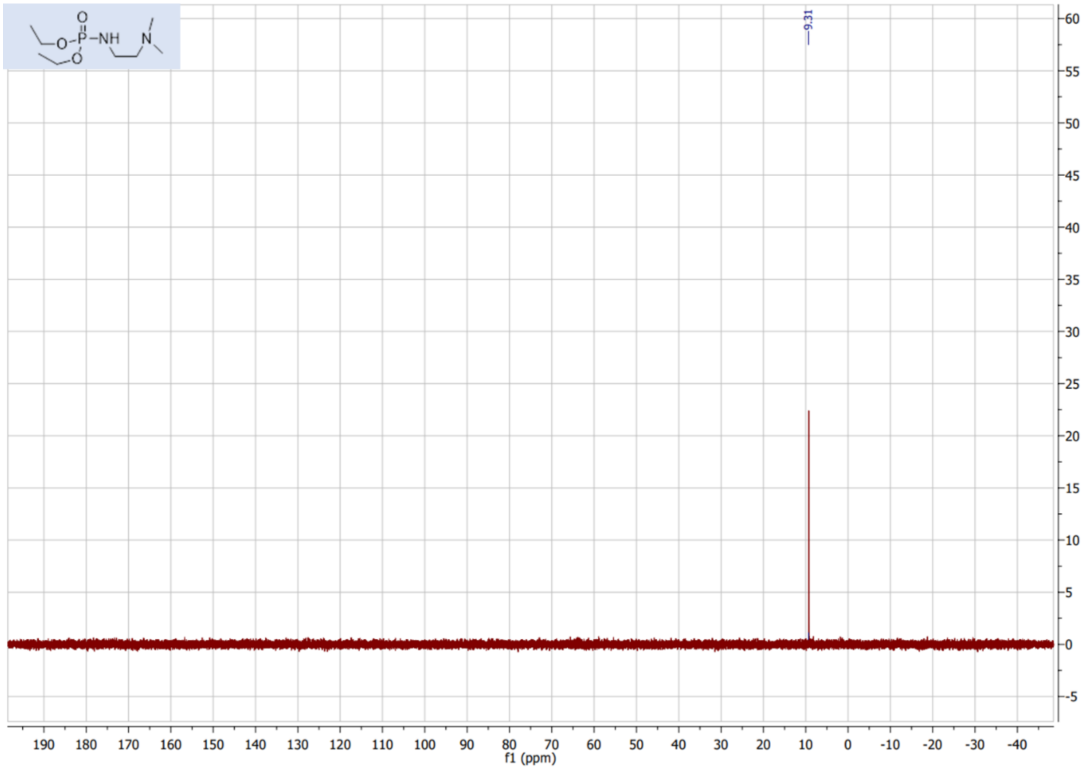

^{31}PNMR spectrum of **P2**

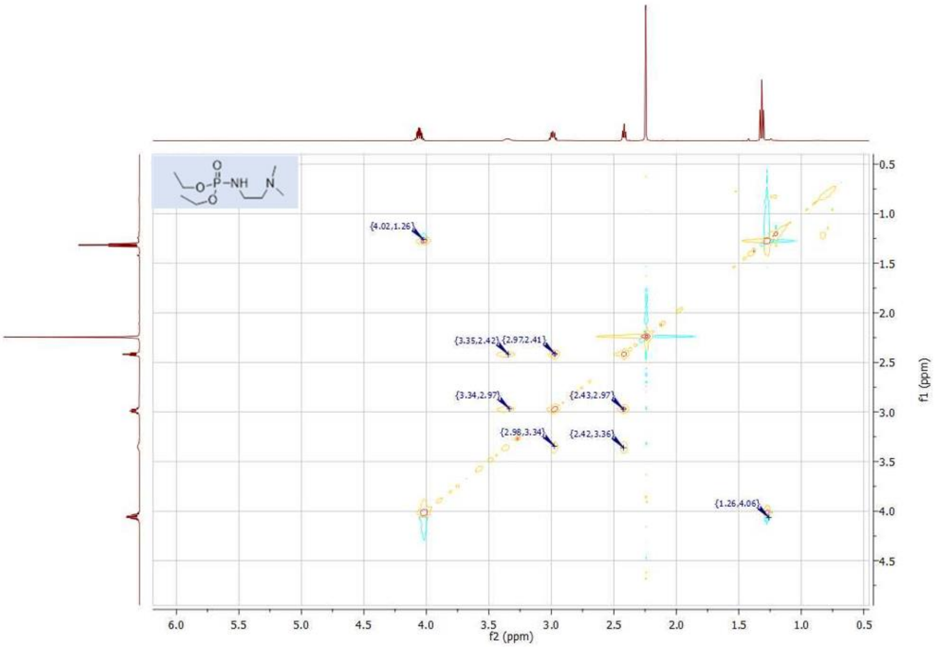

TOCSY spectrum of **P2**

P3

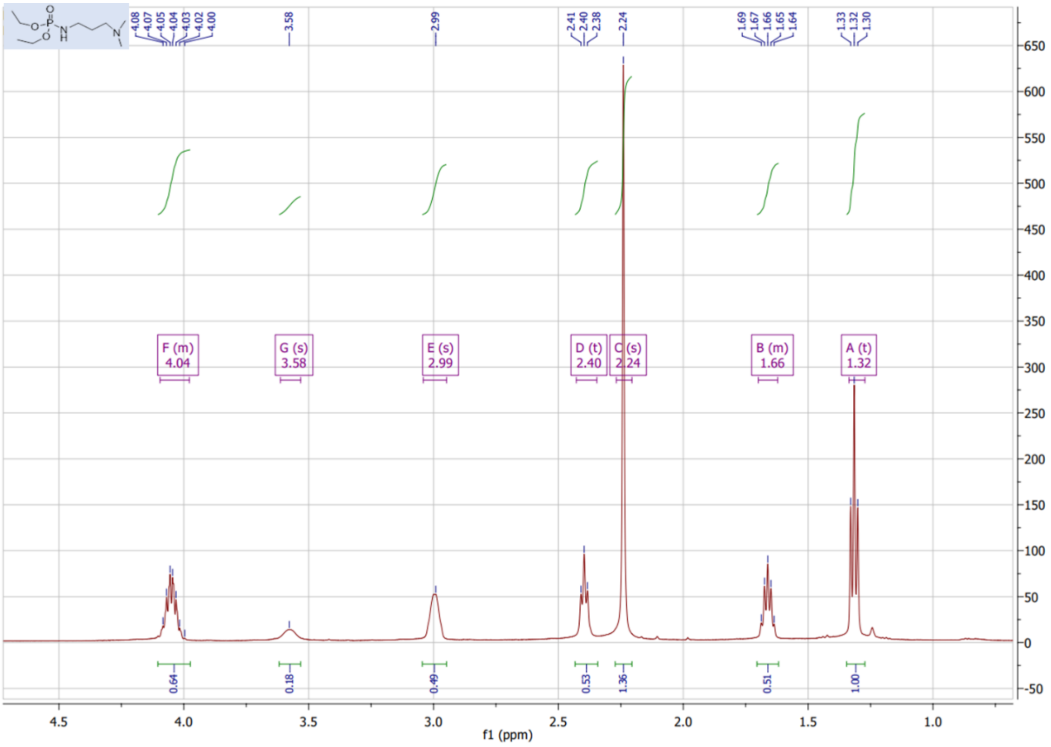

^{1}HNMR spectrum of **P3**

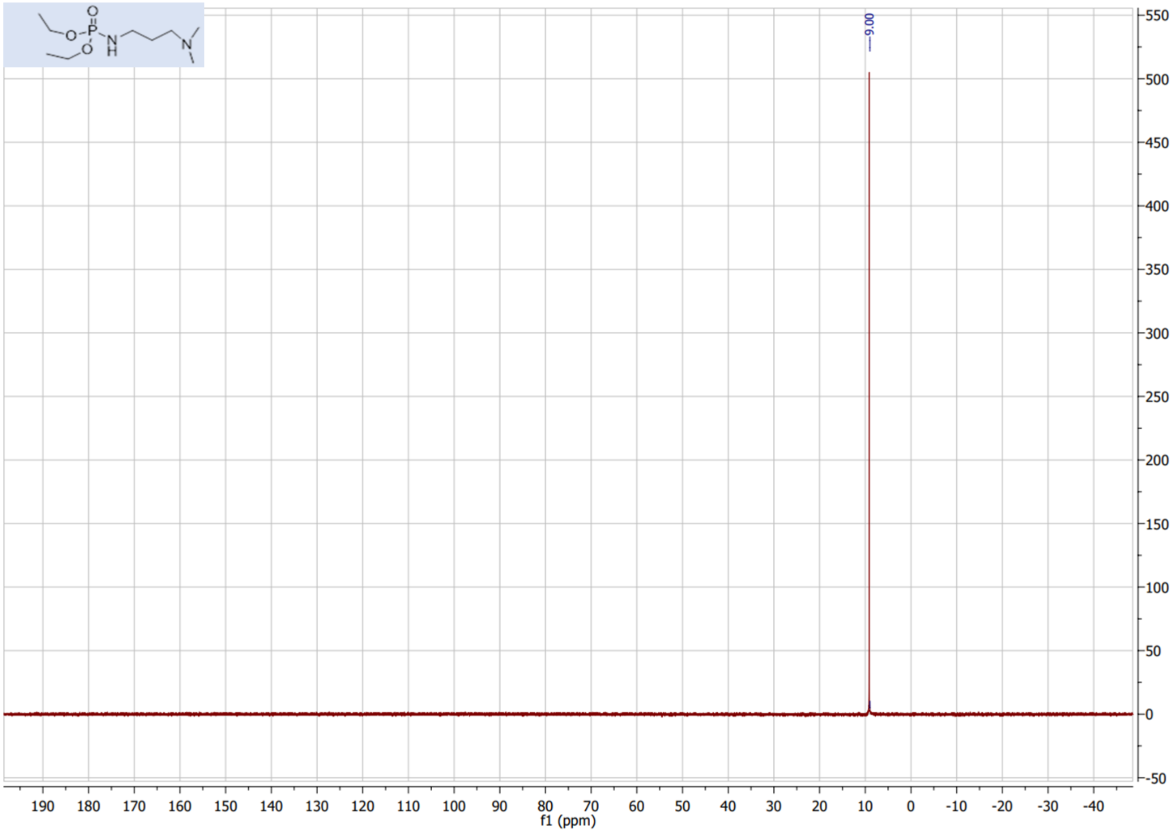

^{31}PNMR spectrum of **P3**

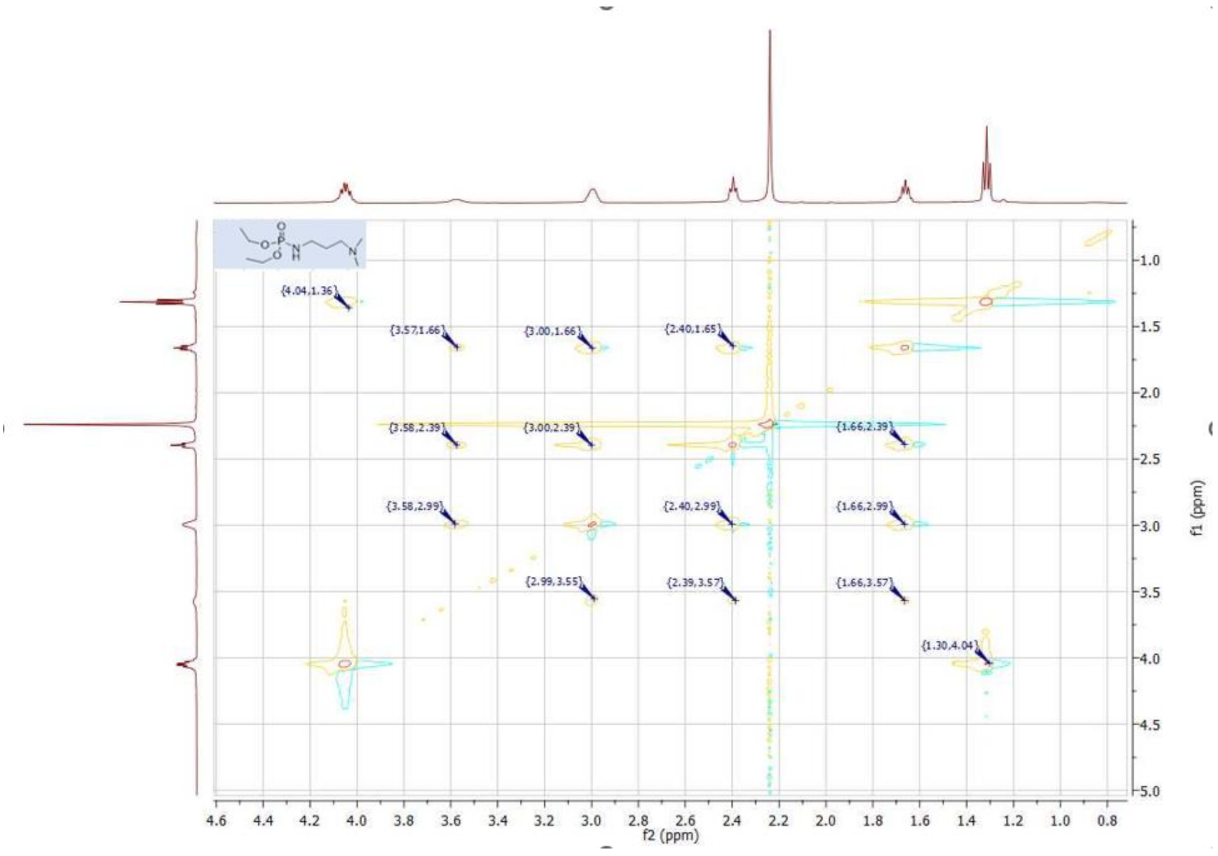

TOCSY spectrum of **P3**

P4

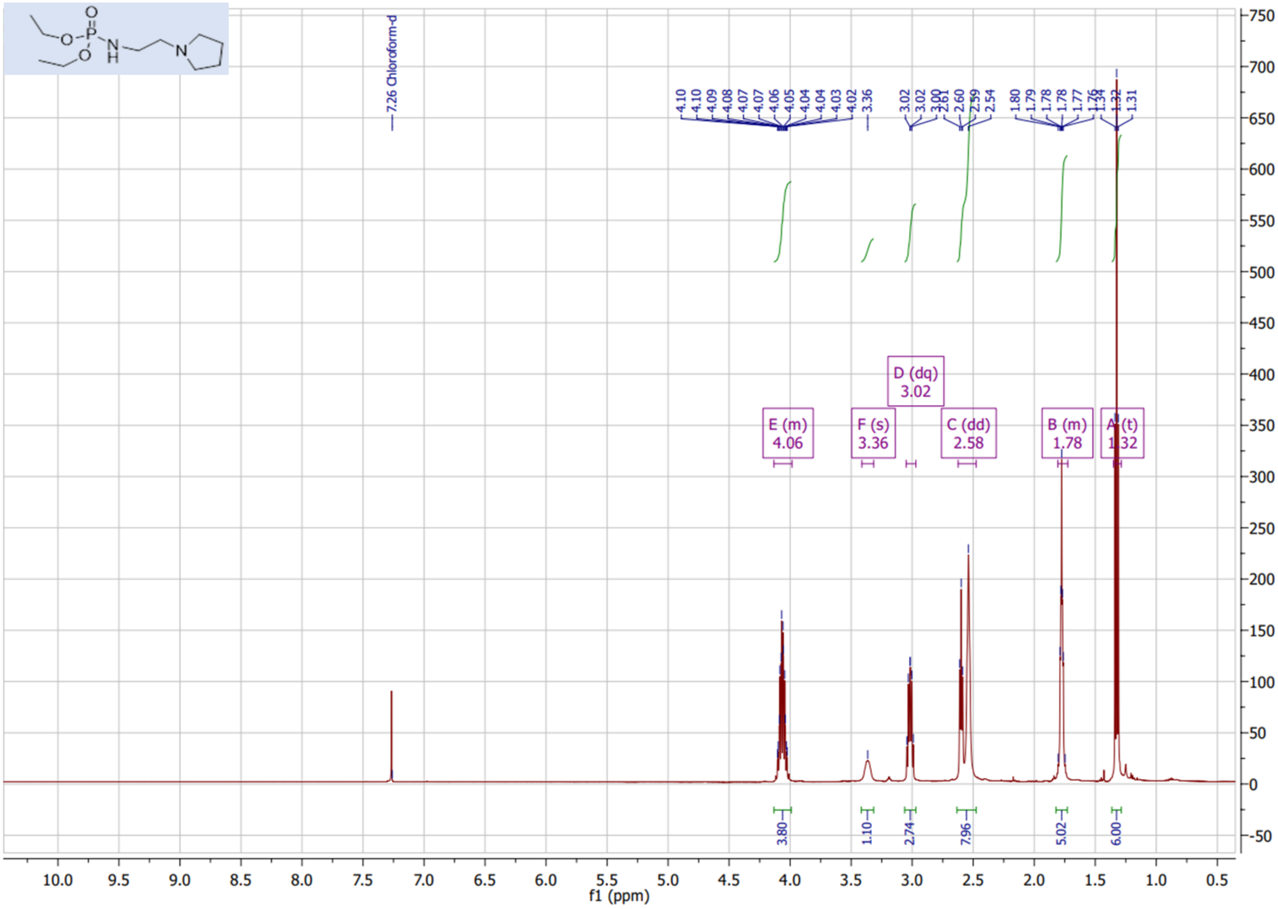

^{1}HNMR spectrum of **P4**

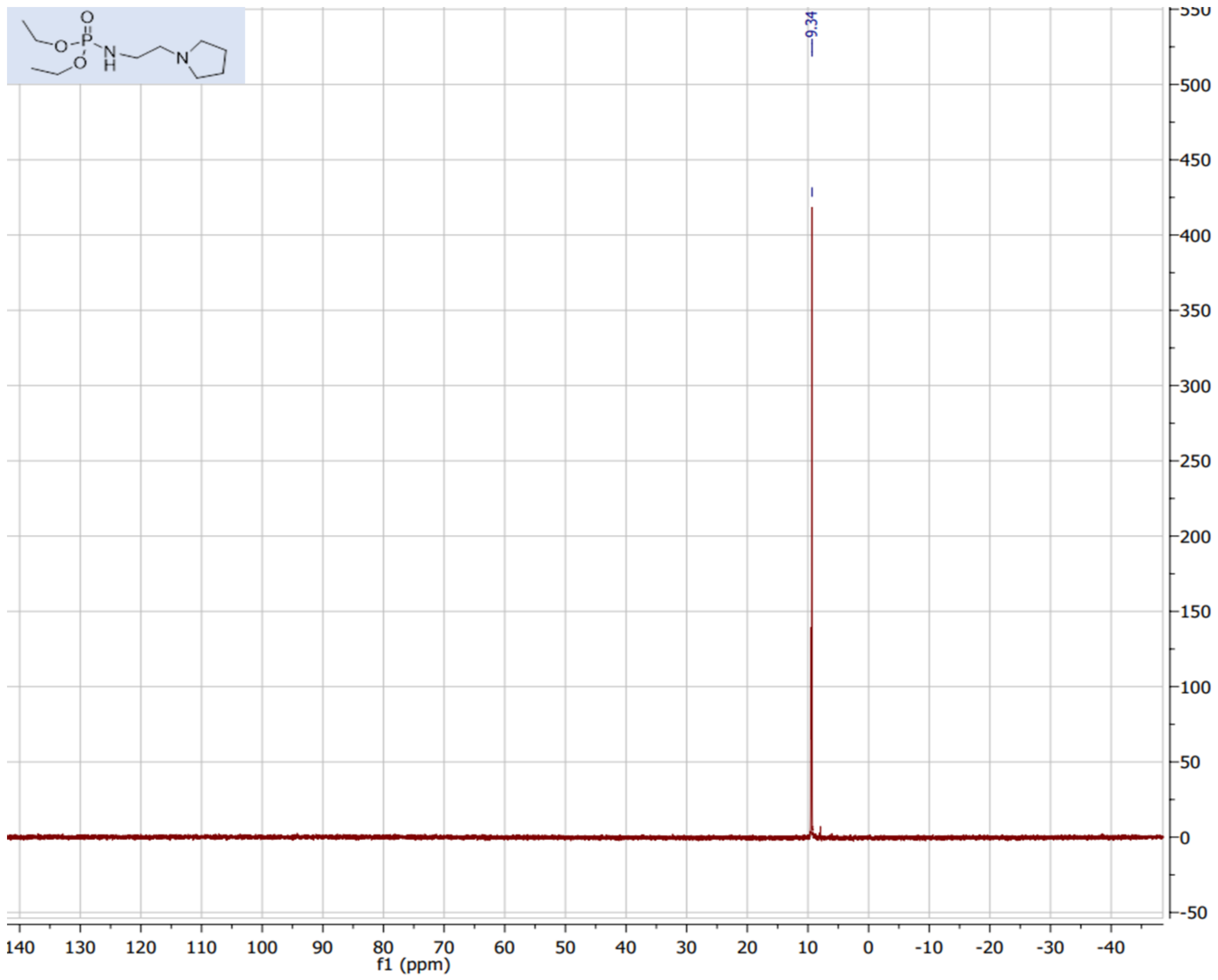

^{31}PNMR spectrum of **P4**

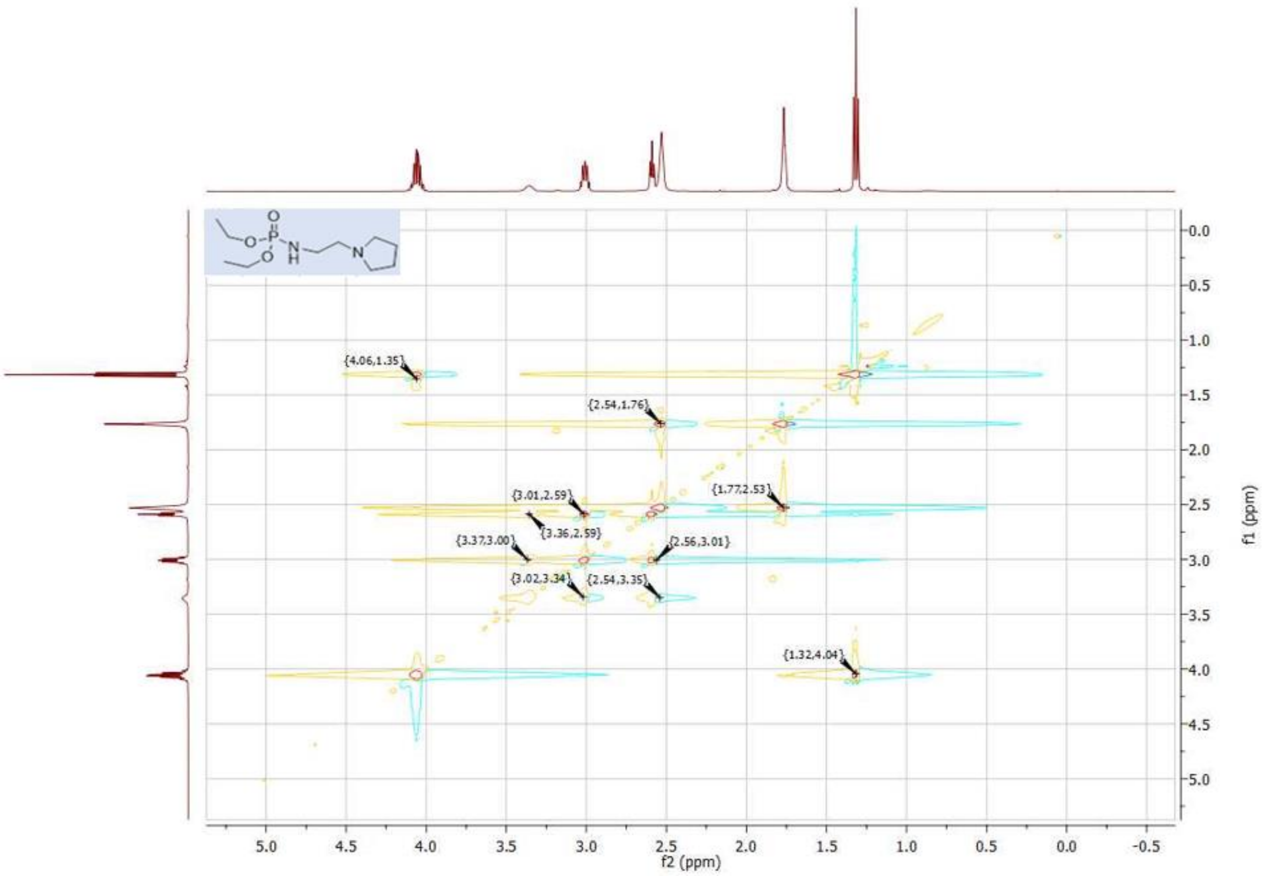

TOCSY spectrum of **P4**
